# ﻿Molecular and morphological evidence for a new species of *Stachys* (Lamiaceae) from Hunan, China

**DOI:** 10.3897/phytokeys.236.112741

**Published:** 2023-12-13

**Authors:** Ling Xue, Jia-Hua Cai, Min Zhan, Xiao-Ping Li, Lei Wu, Ya-Ping Chen

**Affiliations:** 1 College of Forestry, Central South University of Forestry and Technology, Changsha 410004, China Central South University of Forestry and Technology Changsha China; 2 Yingzuijie National Nature Reserve of Hunan Province, Huaihua 563517, China Yingzuijie National Nature Reserve of Hunan Province Huaihua China; 3 CAS Key Laboratory for Plant Diversity and Biogeography of East Asia, Kunming Institute of Botany, Chinese Academy of Sciences, Kunming 650201, China Kunming Institute of Botany, Chinese Academy of Sciences Kunming China

**Keywords:** *Eurystachys* clade, Lamioideae, micromorphology, Stachydeae, taxonomy

## Abstract

*Stachysyingzuijieensis*, a new species from western Hunan, China, is described and illustrated. Molecular phylogenetic analyses based on three nuclear ribosomal DNA loci (ETS, ITS and 5S-NTS) recovered *S.yingzuijieensis* within the *Stachys* clade and as a sister group of *S.arrecta*. The two species can be easily distinguished by the morphology of lamina, corolla and nutlet. A key to all species of Stachydeae from China is also provided.

## ﻿Introduction

As one of the largest genera in Lamiaceae, *Stachys* L. comprises over 365 species distributed worldwide (Bhattacharjee 1980; Harley et al. 2004; POWO 2023). Together with other 11 genera, *Stachys* belongs to the largest tribe in subfamily Lamioideae, i.e. Stachydeae (Zhao et al. 2021). However, the intergeneric relationship within the tribe is taxonomically challenging and *Stachys* has been continuously shown to be non-monophyletic in previous molecular phylogenetic studies. While exploring the phylogenetic position of the Hawaiian endemic mints with respect to *Stachys*, Lindqvist and Albert (2002) showed that three genera endemic to Hawaii (*Haplostachys* (A. Gray) Hillebr., *Phyllostegia* Benth. and *Stenogyne* Benth.), as well as *Prasium* L., *Phlomidoschema* (Benth.) Vved. and *Sideritis* L., were embedded within *Stachys*. Scheen et al. (2010) and Bendiksby et al. (2011) further added the Asian genera *Chamaesphacos* Schrenk ex Fisch. & C.A. Mey., *Hypogomphia* Bunge, *Suzukia* Kudô and *Thuspeinanta* T. Durand to the list of taxa nested within *Stachys* in their lamioid-wide studies. The most comprehensive phylogenetic analyses of Stachydeae were performed by Salmaki et al. (2013, 2019), based on multiple nuclear ribosomal and plastid DNA loci. Salmaki et al. (2019) recognised 12 well-supported clades within the *Eurystachys* clade, a name suggested by Salmaki et al. (2013) to include all genera of Stachydeae, except the monotypic genus *Melittis* L. Though the synapomorphies for Stachydeae remain unclear, members of the tribe usually share campanulate or weakly 2-lipped calyx with spiny lobes and hairy throat, strongly 2-lipped corolla and apically rounded nutlets (Scheen et al. 2010).

A total of 18 species of *Stachys* are recorded from China and eight of them are endemic (Li and Hedge 1994). Except for *Stachys*, China also accommodates another three genera of Stachydeae, i.e. *Chamaesphacos* (1 sp.), *Sideritis* (2 spp.) and *Suzukia* (2 spp.) (Li and Hedge 1994; Liu and Zhang 2004). Recently, a potential new species of *Stachys* was discovered during our field investigation in western Hunan Province, China. By carrying out comprehensive molecular phylogenetic and morphological studies, we confirmed its status as a species new to science. It was, thus, named *Stachysyingzuijieensis* L. Wu & Y.P. Chen and described below.

## ﻿Materials and methods

### ﻿Molecular phylogenetic analyses

The phylogenetic placement of the new species within Stachydeae was evaluated based on the framework of Salmaki et al. (2019). A total of 90 accessions representing 88 taxa from all 12 clades and 11 genera of *Eurystachys*, as well as *Melittismelissophyllum* L., were sampled as the ingroups. Two genera that are closely related to Stachydeae – *Betonica* L. and *Galeopsis* L. – were selected as the outgroups. Except for one accession of the new species and one accession for each of eight species of *Stachys* from China that were newly sequenced here, all remaining sequences were downloaded from GenBank. Voucher information for newly-sequenced samples and GenBank accession numbers for all sequences are listed in Appendix 1.

Total genomic DNA was extracted from silica-gel-dried leaf material using the modified CTAB method (Doyle and Doyle 1987). According to Salmaki et al. (2019), three nuclear ribosomal DNA loci, i.e. the internal and external transcribed spacers (ITS and ETS) and the 5S non-transcribed spacer (5S-NTS), were used to reconstruct the phylogenetic relationships. Polymerase chain reaction primers and protocols of ITS and ETS followed those used by Chen et al. (2019) and that of 5S-NTS followed Roy et al. (2013).

Raw sequences were assembled and edited using Geneious v.11.0.3 (Kearse et al. 2012). Data matrices were aligned using MUSCLE (Edgar 2004) and then manually adjusted in Geneious. After removing the ambiguously aligned regions in the ITS dataset, the three DNA loci were concatenated for phylogenetic reconstruction. Partitioned Bayesian Inference (BI) and partitioned Maximum Likelihood (ML) analyses were performed on the web server Cyberinfrastructure for Phylogenetic Research Science (CIPRES) Gateway (http://www.phylo.org/; Miller et al. 2010), using RAxML-HPC2 (Stamatakis 2014) and MrBayes v.3.2.2 (Ronquist et al. 2012), respectively. Detailed settings for the two analyses followed those described in Chen et al. (2019). The resulting trees were visualised in TreeGraph 2 (Stover and Müller 2010).

### ﻿Morphological studies

Morphological similarities and differences between the new species and other taxa of Stachydeae were compared, based on our previous field investigations and specimen examination. Images of specimens (including type specimens) and living plants of Stachydeae from JSTOR (https://www.jstor.org/), Global Biodiversity Information Facility (GBIF, https://www.gbif.org/), Chinese Virtual Herbarium (CVH, https://www.cvh.ac.cn/) and Plant Photo Bank of China (PPBC, http://ppbc.iplant.cn/) were examined. Protologues and other taxonomic and floristic literature related to Stachydeae (Knorring 1954; Ball 1972; Nelson 1981; Bhattacharjee 1982; Codd 1985; Li and Hedge 1994; Turner 1994; Paton et al. 2009; Salmaki et al. 2012) was also reviewed.

Trichomes on the lamina and calyx, as well as the nutlet and pollen morphology of the new species, were investigated using scanning electron microscopy (SEM). All materials were directly mounted on to stubs and sputter-coated with gold for 90 s at 20 mA. Micromorphological observations were conducted using a Zeiss EVO LS10 scanning electron microscope (Carl Zeiss NTS, Oberkochen, Germany) at 10 kV. Terminologies used for trichome, nutlet and pollen description followed those of Salmaki et al. (2008a, 2008b, 2009), Karaismailoğlu and Güner (2019, 2021) and Totmaj and Salmaki (2022).

## ﻿Results

### ﻿Phylogenetic results

**Figure 1.**
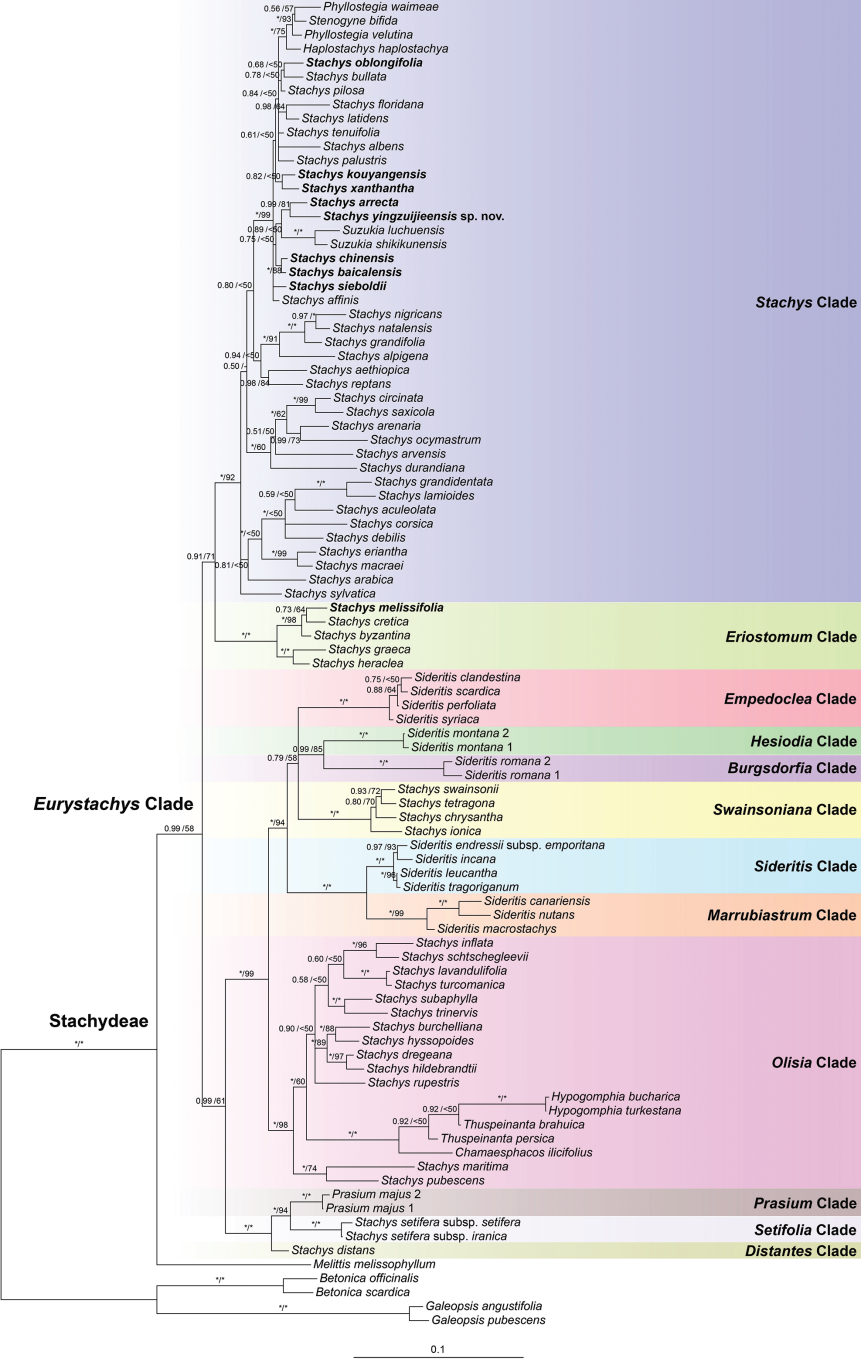
Bayesian 50% majority-rule consensus tree of Stachydeae based on combined nuclear (ITS, ETS and 5S-NTS) dataset. Support values ≥ 0.50 PP or 50% BS are displayed above the branches (an “*” indicates a support value = 1.00 PP or 100% BS and a “-” indicates a conflicting node in the BI and ML trees). Species marked in bold represent samples newly sequenced in the present study. Multiple accessions of the same species are numbered according to Appendix 1.

The aligned length of the combined nuclear dataset was 1,381 bp (589 bp for ITS, 456 bp for ETS and 336 bp for 5S-NTS). The topologies of the BI and ML trees were largely consistent with each other, but the BI tree provided higher resolution. Thus, only the Bayesian 50% majority-rule consensus tree was presented (Fig. 1), the posterior probabilities (PP) and Bootstrap support (BS) values being superimposed on the nodes.

Our molecular phylogenetic result (Fig. 1) revealed that *Melittis* was sister to the remaining Stachydeae, i.e. the *Eurystachys* clade (PP = 0.99, BS = 58%). Two large clades were resolved within the *Eurystachys* clade: the first one (PP = 0.91, BS = 71%) mainly included temperate North American, Hawaiian and several Old World taxa and the second one (PP = 0.99, BS = 61%) only comprised Old World (mostly Mediterranean) taxa. Twelve robustly supported small clades (PP = 1.00, BS > 90%) can be further recognised, with two clades (*Eriostomum* clade and *Stachys* clade) in the first *Eurystachys* clade and the remaining (*Burgsdorfia* clade, *Distantes* clade, *Empedoclia* clade, *Hesiodia* clade, *Marrubiastrum* clade, *Olisia* clade, *Prasium* clade, *Setifolia* clade, *Sideritis* clade and *Swainsoniana* clade) in the second *Eurystachys* clade. Species distributed in China were mostly recovered in the *Stachys* clade, including the new species. Although relationships within the *Stachys* clade were poorly resolved, *Stachysyingzuijieensis* was strongly supported as sister to *Stachysarrecta* L.H. Bailey (PP = 0.99, BS = 81%).

### ﻿Morphological results

**Figure 2.**
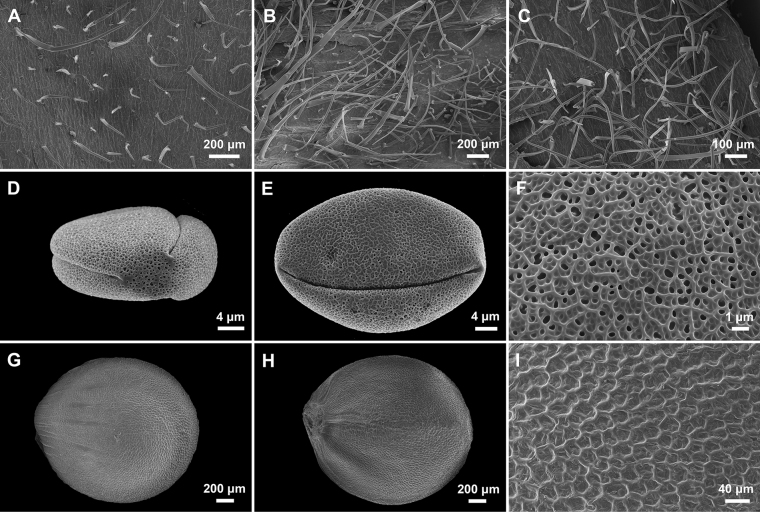
Trichome, pollen, and nutlet micromorphology of *Stachysyingzuijieensis***A** trichomes on the adaxial surface of lamina **B** trichomes on the abaxial surface of lamina **C** trichomes on the outside surface of calyx **D** polar view of pollen **E** equatorial view of pollen **F** surface sculpturing of pollen **G** dorsal view of nutlet **H** ventral view of nutlet **I** surface sculpturing of nutlet.

Stalked glandular and simple non-glandular trichomes were found on both surfaces of the lamina as well as the calyx of the new species (Fig. 2A–C). The abaxial surface of lamina and the outside surface of the calyx were more densely covered with longer trichomes. Pollen grains of *Stachysyingzuijieensis* were tricolpate with reticulate exine sculpturing (Fig. 2D–F), while nutlets were ovate with glabrous and reticulate surface (Fig. 2G–I).

## ﻿Discussion

The backbone of Stachydeae in the present study and the 12 clades recovered in the *Eurystachys* clade (Fig. 1) were consistent with that of Salmaki et al. (2019). The *Stachys* clade, which was referred to as the “*Stachys* core clade” in Salmaki et al. (2013) and the *Stachys* s.s. clade in Lindqvist and Albert (2002), is one of the largest monophyletic groups in the *Eurystachys* clade and comprises five genera (*Haplostachys*, *Phyllostegia*, *Stachys*, *Stenogyne* and *Suzukia*) and over 100 species. No synapomorphy has been found for this clade due to large morphological and geographical diversity (Salmaki et al. 2019). Next-generation sequencing data and comprehensive morphological studies are needed to further clarify the synapomorphies and relationships within this taxonomically problematic and important group.

Only several representatives of Stachydeae from China had been included in previous molecular phylogenetic studies and no morphological study had been carried out for Chinese *Stachys*. In this study, nine species of *Stachys* from China were newly sequenced and included in the phylogenetic analyses. Our results showed that most species that were collected from or reported to be occurring in China were recovered within the *Stachys* clade, including the new species (Fig. 1). *Stachysyingzuijieensis* was further revealed to be sister to *Stachysarrecta*, a species distributed in the evergreen broad-leaved forests at altitudes of 1500–2000 m in central China.

**Figure 3.**
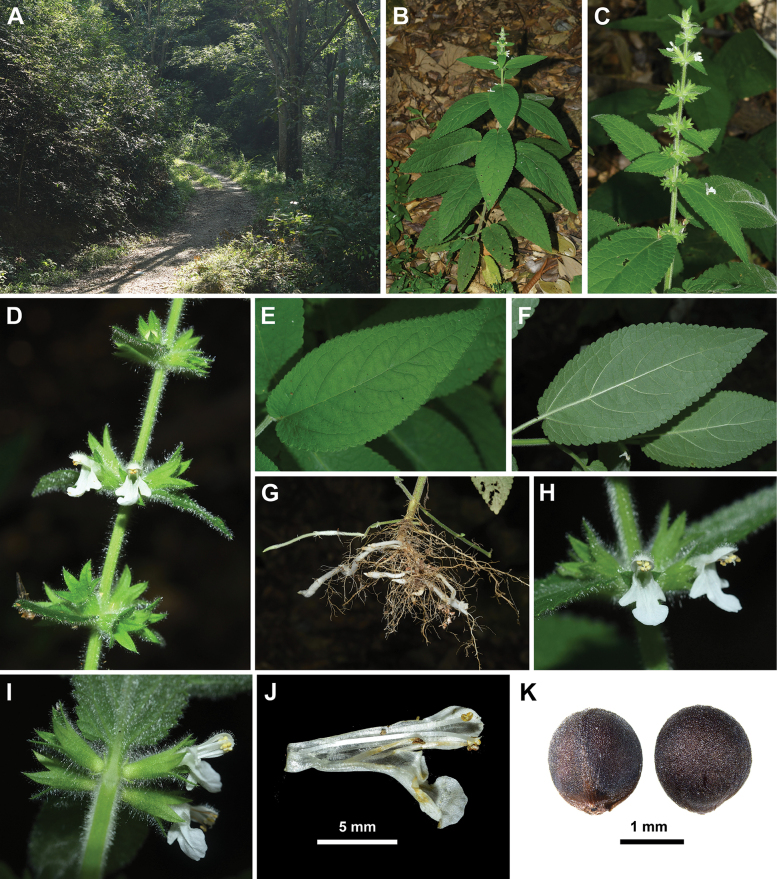
Morphology of *Stachysyingzuijieensis* from the type locality **A** habitat **B** habit **C–D** inflorescence **E** adaxial view of lamina **F** abaxial view of lamina **G** roots and rhizomes **H** frontal view of corolla **I** lateral view of calyces **J** dissected corolla **K** nutlets (**A–J** photographed by Lei Wu **K** photographed by Ya-Ping Chen).

**Figure 4.**
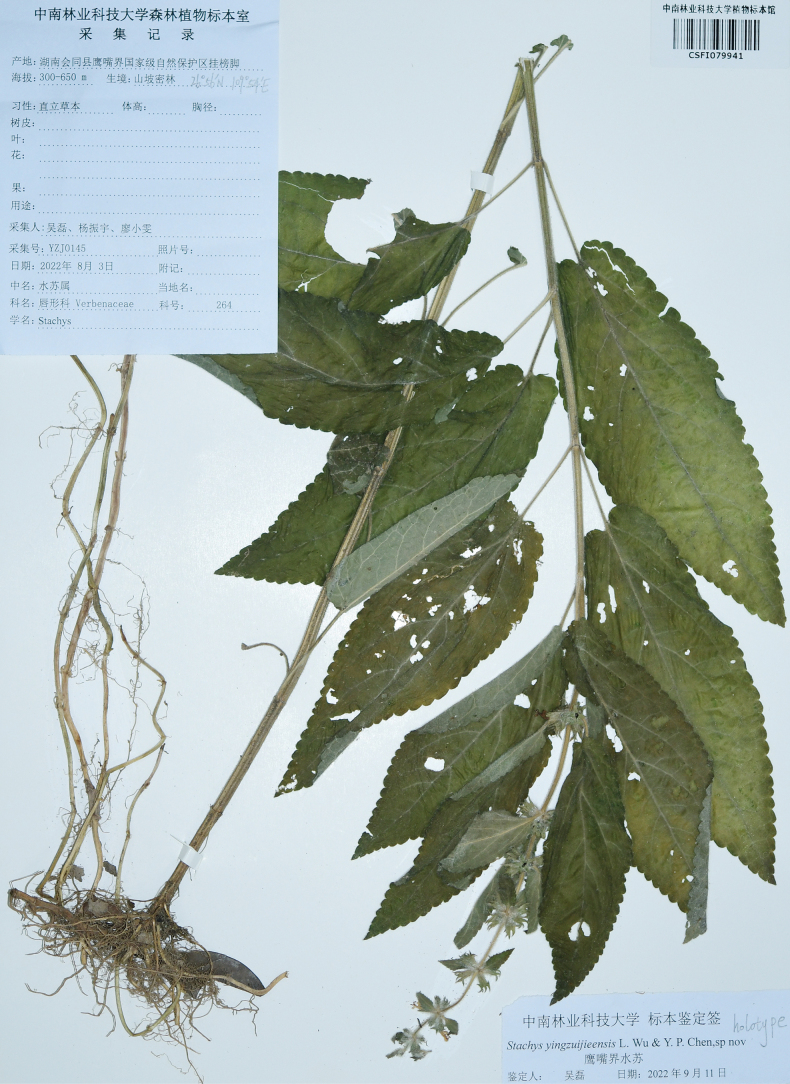
Holotype specimen of *Stachysyingzuijieensis*.

*Stachysyingzuijieensis* differs from all other Chinese Stachydeae in its densely villosus and glandular pubescent plants, as well as the white corollas with tube included in the calyces (Figs 3, 4). For example, the corollas of *Stachysarrecta* are pink with purple spots and the corolla tubes are exerted from the calyces (Li and Hedge 1994). Except for above differences, *Stachysyingzuijieensis* also has oblong to oblong-lanceolate laminae with crenulate margin, whereas the laminae of *Stachysarrecta* are cordate with coarsely serrate margin. Moreover, they can be distinguished in the nutlet surface, which is smooth in the new species (Fig. 2), but verrucate in *Stachysarrecta*. More detailed differences between the two species are listed in Table 1. Here, we also provided a key to all species of Stachydeae from China below.

**Table 1.** Morphological comparisons between *Stachysyingzuijieensis* and *S.arrecta*.

Characters	*S.yingzuijieensis*	*S.arrecta*
Lamina	Oblong to oblong-lanceolate, 10–16 × 4–6 cm, margin crenulate	Cordate, 2.5–6.5 × 1.5–3 cm, margin coarsely serrate
Calyx	Approximately 7 mm long, teeth ca. 3 mm long, ovate-lanceolate	Approximately 5 mm long, teeth 2–2.5 mm long, narrowly triangular
Pedicel	Absent	Approximately 1 mm long
Corolla	White without spots, ca. 1 cm long, tube included in calyx	Pink with purple spots, ca. 1.2 cm long, tube exerted from calyx
Nutlet	Surface smooth	Surface verrucate

### ﻿Key to the species of Stachydeae from China

**Table:** 

1	Creeping herbs	**2**
–	Erect herbs	**3**
2	Middle lobe of lower corolla lip entire	***Suzukiashikikunensis***
–	Middle lobe of lower corolla lip irregularly incised	***Suzukialuchuensis***
3	Lamina spinescent-aristate	***Chamaesphacosilicifolius***
–	Lamina not spinescent-aristate	**4**
4	Calyx tubular-campanulate; corolla included in calyx	**5**
–	Calyx campanulate; corolla exserted from calyx	**6**
5	Corolla yellow, middle lobe of lower lip incised	***Sideritismontana***
–	Corolla purple, middle lobe of lower lip entire	***Sideritisbalansae***
6	Annual herbs	***Stachysarvensis***
–	Perennial herbs	**7**
7	Bracteoles over half as long as calyx	**8**
–	Bracteoles less than half as long as calyx, early deciduous	**9**
8	Plants densely sericeous-lanate; verticillasters in compact spikes	***Stachyslanata***
–	Plants pilose; verticillasters in widely spaced spikes	***Stachysmelissifolia***
9	Lamina oblong, lanceolate to oblong-lanceolate	**10**
–	Lamina ovate, ovate-oblong, or cordate	**16**
10	Corolla white	***Stachysyingzuijieensis***
–	Corolla pink, purple to red-purple	**11**
11	Lamina densely villous-tomentose abaxially	***Stachysoblongifolia***
–	Lamina hispid, puberulent, or glabrous abaxially	**12**
12	Stems densely retrorse villous	***Stachyspalustris***
–	Stems spreading hispid, glabrous, or subglabrous	**13**
13	Calyx densely villous-hispid outside	***Stachysbaicalensis***
–	Calyx sparsely villous-hispid or glandular puberulent outside	**14**
14	Calyx teeth obtuse at apex; corolla tube long exserted from calyx	***Stachysadulterina***
–	Calyx teeth spinescent at apex; corolla tube included in calyx	**15**
15	Lamina sparsely minutely hispid or subglabrous adaxially; calyx sparsely villous-hispid along veins outside	***Stachyschinensis***
–	Lamina glabrous adaxially; calyx glandular puberulent outside	***Stachysjaponica***
16	Corolla white or yellow	**17**
–	Corolla pink or purple	**18**
17	Corolla white; calyx teeth triangular, less than 2 mm long	***Stachystaliensis***
–	Corolla yellow; calyx teeth ovate-triangular, over 2 mm long	***Stachysxanthantha***
18	Rhizomes not enlarged or succulent	**19**
–	Rhizomes enlarged, succulent	**21**
19	Lamina over 8 cm long	***Stachyssylvatica***
–	Lamina less than 5 cm long	**20**
20	Calyx teeth ovate-lanceolate	***Stachysstrictiflora***
–	Calyx teeth triangular	***Stachyskouyangensis***
21	Calyx teeth linear-lanceolate, reflexed	***Stachyspseudophlomis***
–	Calyx teeth narrowly triangular to triangular, straight	**22**
22	Lamina ovate-oblong; nutlet smooth	***Stachysgeobombycis***
–	Lamina ovate to cordate; nutlet tuberculate	**23**
23	Lamina cordate; calyx ca. 5 mm long	***Stachysarrecta***
–	Lamina ovate to oblong-ovate; calyx ca. 9 mm long	***Stachyssieboldii***

## ﻿Taxonomic treatment

### ﻿ Stachys yingzuijieensis

Taxon classificationPlantaeLamialesLamiaceae

L.Wu & Y.P.Chen sp. nov.

0BE63CBF-2ADC-54F5-8B05-236C5AF00F27

urn:lsid:ipni.org:names:77332759-1

Figs 3 , 4

#### Type.

China, Hunan, Huitong County, Yingzuijie National Nature Reserve, alt. 300–800 m, 26°56′N, 109°54′E, 3 Aug 2022, L. Wu et al. YZJ0145 (holotype: CSFI079941!; isotype: CSFI!).

#### Diagnosis.

*Stachysyingzuijieensis* is most closely related to *S.arrecta*, but differs in its lamina oblong to elliptic-lanceolate (vs. cordate) with margin crenulate (vs. coarsely serrate), corolla white (vs. pink with purple spots) with tube included in calyx (vs. exerted from calyx) and nutlet surface smooth (vs. verrucate).

Herbs perennial. Rhizomes white, densely glandular pubescent. Stems erect, simple, 50–75 cm long, quadrangular, densely villous and glandular pubescent. Leaves opposite; petioles 2–4 cm long, densely villous and glandular pubescent; lamina oblong to oblong-lanceolate, papery, 10–16 × 4–6 cm, apex acute, margin crenulate, base cordate, adaxially green, sparsely villous and glandular pubescent, abaxially light green, densely villous and glandular pubescent, lateral veins 4–5-paired, conspicuously elevated abaxially. Verticillasters 6-flowered, flowers sessile; bracts leaf-like, upper ones sessile, lanceolate, densely villous and glandular pubescent on both surfaces, longer than verticillasters; bracteoles linear, 1–2 mm long. Calyx campanulate, ca. 7 mm long, 10-veined, densely villous and glandular pubescent outside, glandular pubescent inside, fruiting calyx dilated, ca. 9 mm long; teeth 5, subequal, ovate-lanceolate, ca. 3 mm long, apex spinescent. Corolla white, ca. 1 cm long, tube ca. 7 mm long, ca. 1.5 mm wide, pubescent annulate inside at 1/3 distance from base; 2-lipped, upper lip erect, concave, subcircular, ca. 3 mm in diam., densely pubescent and glandular pubescent outside, glabrous inside, lower lip spreading, sparsely pubescent and glandular pubescent to glabrescent outside, glabrous inside, ca. 6 mm long, 3-lobed, medium lob largest, trapeziform, ca. 3 mm long, ca. 4 mm wide, apex entire or emarginate, lateral lobs oblong, ca. 2 mm long, ca. 1 mm wide. Stamens 4, straight, included, filaments pubescent and glandular pubescent, anther cells 2, divergent. Style included, glabrous, apex subequally 2-lobed, lobes subulate. Ovary rounded at apex, glabrous. Nutlets 4, dark brown, ovoid, ca. 1.5 mm in diam., smooth and glabrous.

#### Phenology.

Flowering from July to September, fruiting from August to October.

#### Distribution and habitat.

Currently, *S.yingzuijieensis* is only known from the Yingzhuijie National Nature Reserve and a total of 50 mature plants were found during our field investigation. The new species usually grows in shady and moist places in evergreen broad-leaved forests at an altitude of 300–800 m.

#### Etymology.

The specific epithet is derived from the type locality of the new species, i.e. the Yingzuijie National Nature Reserve in western Hunan Province, China.

#### Chinese name (assigned here).

yīng zhuǐ jiè shuǐ sū (鹰嘴界水苏).

#### Additional specimen examined.

China. Hunan: Huitong County, Yingzuijie National Nature Reserve, 8 Aug 2022, L. Wu et al. YZJ0654 (CSFI!).

## Supplementary Material

XML Treatment for Stachys yingzuijieensis

## ﻿Additional information

### Author ORCIDs

Ya-Ping Chen https://orcid.org/0000-0002-7502-1848

### Data availability

All of the data that support the findings of this study are available in the main text.

## ﻿Appendix 1

Specimen information (taxon, voucher, herbarium, country) for samples newly sequenced in the present study with GenBank accession numbers for ITS, ETS and 5S-NTS, respectively. A “-” indicates a missing sequence. Herbarium abbreviations are listed after the vouchers. The accession numbers marked with an asterisk represent sequences newly generated. Only GenBank accession numbers are listed for sequences downloaded from NCBI.

***Betonicaofficinalis*** L., KF529533, MK909580, MK909684; ***Betonicascardica*** Griseb., KF529534, MK909581, -; ***Chamaesphacosilicifolius*** Schrenk ex Fisch. & C.A. Mey., KF529540, MK909585, MK909689; ***Galeopsisangustifolia*** Ehrh. ex Hoffm, KF529535, -, MK909685; ***Galeopsispubescens*** Besser, KF529536, -, MK909686; ***Haplostachyshaplostachya*** (A. Gray) H.St. John, KF529541, -, MK909690; ***Hypogomphiabucharica*** Vved., KF529542, MK909587, MK909691; ***Hypogomphiaturkestana*** Bunge, KF529543, MK909588, MK909692; ***Melittismelissophyllum*** L., KF529544, KF235787, MK909693; ***Phyllostegiavelutina*** (Sherff) H.St. John, KF549547, KF235809, AF308212; ***Phyllostegiawaimeae*** Wawra, KF529548, KF235811, KF235752; ***Prasiummajus*** L. 1, KF529549, MK909590, MK909694; ***Prasiummajus*** L. 2, KF529550, MK909591, AF501919; ***Sideritiscanariensis*** L., AF335605, MK909593, MK909695; ***Sideritisclandestina*** (Bory & Chaub.) Hayek, AF335616, MK909595, -; **Sideritisendressiisubsp.emporitana** Willk, AF335627, MK909598, MK909698; ***Sideritisincana*** L., AF335634, MK909601, -; ***Sideritisleucantha*** Cav., AF335636, MK909603, MK909700; ***Sideritismacrostachys*** Poir., AF335609, -, AF501920; ***Sideritismontana*** L. 1, AF335612, -, MK909701; ***Sideritismontana*** L. 2, KF529551, -, MK909702; ***Sideritisnutans*** Svent., DQ900767, MK909604, MK909703; ***Sideritisperfoliata*** L., AF335618, -, MK909705; ***Sideritisromana*** L. 1, AF335614, -, MK909706; ***Sideritisromana*** L. 2, KF529552, -, AF501922; ***Sideritisscardica*** Griseb., AF335619, MK909606, MK909707; ***Sideritissyriaca*** L., AF335620, -, MK909708; ***Sideritistragoriganum*** Lag., AF335639, MK909608, MK909710; ***Stachysaculeolata*** Hook. f., KF529556, KF235814, AF501924; ***Stachysaethiopica*** L., KF529559, KF235815, KF235753; ***Stachysaffinis*** Bunge, MH703287, KF235816, AF501925; ***Stachysalbens*** A. Gray, KF529560, MK909613, AF501928; ***Stachysalpigena*** T.C.E. Fr., KF529561, KF235822, KF235755; ***Stachysarabica*** Hornem., KF529564, MK909616, MK909711; ***Stachysarenaria*** Vahl, KF529566, MK909618, -; ***Stachysarrecta*** L.H. Bailey, H.J. Dong et al. HGNU-0485 (KUN), Hubei, China, OR878465*, OR887616*, OR887626*; ***Stachysarvensis*** (L.) L., KF529567, MK909619, MK909712; ***Stachysbaicalensis*** Fisch. ex Benth., Y.P. Chen & Y. Zhao EM1459 (KUN), Hebei, China, OR878468*, OR887619*, OR887629*; ***Stachysbullata*** Benth., KF529576, KF235831, KF235759; ***Stachysburchelliana*** Launert, KF529574, MK909624, MK909713; ***Stachysbyzantina*** K. Koch, KF529577, KF235832, AF501938; ***Stachyschinensis*** Bunge ex Benth., B. Liu et al. 7252 (PE), Beijing Botanical Garden (cultivated), China, OR878467*, OR887618*, OR887628*; ***Stachyschrysantha*** Boiss. & Heldr., KF529580, MK909628, AF501939; ***Stachyscircinata*** L’Hér., KF529581, MK909629, -; ***Stachyscorsica*** Pers., KF529582, KF235836, KF235762; ***Stachyscretica*** L., KF529583, KF235838, AF501948; ***Stachysdebilis*** Kunth, KF529584, KF235839, KF235763; ***Stachysdistans*** Benth., KF529585, MK909630, MK909716; ***Stachysdregeana*** Benth., KF529586, MK909631, MK909717; ***Stachysdurandiana*** Coss., KF529587, MK909632, MK909718; ***Stachyseriantha*** Benth., KF529589, KF235842, AF501951; ***Stachysfloridana*** Shuttlew. ex Benth, KF529590, KF235843, AF501952; ***Stachysgraeca*** Boiss. & Heldr., KF529595, MK909636, MK909719; ***Stachysgrandidentata*** Lindl., KF529596, KF235845, KF235766; ***Stachysgrandifolia*** E. Mey., KF529597, MK909637, -; ***Stachysheraclea*** All., KF529598, MK909638, MK909720; ***Stachyshildebrandtii*** Vatke, KF529599, MK909639, MK909721; ***Stachyshyssopoides*** Burch. ex Benth, KF529600, MK909640, MK909722; ***Stachysinflata*** Benth., KF529601, MK909641, MK909723; ***Stachysionica*** Halácsy, KF529602, MK909642, MK909724; ***Stachyskouyangensis*** (Vaniot) Dunn, Y.P. Chen et al. EM482 (KUN), Yunnan, China, OR878463*, OR887614*, OR887624*; ***Stachyslamioides*** Benth., KF529607, KF235852, KF235773; ***Stachyslatidens*** Small, KF529608, KF235854, AF501956; ***Stachyslavandulifolia*** Vahl, KF529609, MK909646, MK909725; ***Stachysmacraei*** Benth., KF529611, KF235857, KF235774; ***Stachysmaritima*** Gouan, KF529612, MK909647, MK909726; ***Stachysmelissifolia*** Benth., Y.P. Chen et al. EM1206 (KUN), Xizang, China, OR878466*, OR887617*, OR887627*; ***Stachysnatalensis*** Hochst., KF529619, MK909654, -; ***Stachysnigricans*** Benth., KF529622, MK909657, -; ***Stachysoblongifolia*** Wall. ex Benth., H. Peng et al. FJ881 (KUN), Guizhou, China, OR878472*, OR887623*, OR887632*; ***Stachysocymastrum*** (L.) Briq., KF529623, MK909658, MK909727; ***Stachyspalustris*** L., KF529624, MK909659, MK909728; ***Stachyspilosa*** Nutt., KF529628, KF235861, MK909730; ***Stachyspubescens*** Ten., KF529629, MK909663, MK909731; ***Stachysreptans*** Hedge, KF529632, MK909664, MK909732; ***Stachysrupestris*** Montbret & Aucher ex Benth., KF529633, MK909665, MK909733; ***Stachyssaxicola*** Coss. & Balansa, KF529634, MK909666, MK909734; ***Stachysschtschegleevii*** Sosn. ex Grossh., KF529637, MK909667, MK909736; **Stachyssetiferasubsp.iranica** (Rech. f.) Rech. f., KF529636, -, MK909735; **Stachyssetiferasubsp.setifera** C.A. Mey., KF529635, -, AF501976; ***Stachyssieboldii*** Miq., Y.P. Chen & Y. Zhao EM1492 (KUN), Ningxia, China, OR878469*, OR887620*, OR887630*; ***Stachyssubaphylla*** Rech.f., KF529641, MK909671, MK909737; ***Stachysswainsonii*** Benth., KF529642, KF235871, AF501977; ***Stachyssylvatica*** L., KF529643, MK909672, MK909738; ***Stachystenuifolia*** Willd., OR392565, -, AF501981; ***Stachystetragona*** Boiss. & Heldr., KF529646, MK909673, MK909739; ***Stachystrinervis*** Aitch. & Hemsl., KF529647, MK909674, MK909740; ***Stachysturcomanica*** Trautv., KF529649, MK909676, MK909742; ***Stachysxanthantha*** C.Y. Wu, Y.P. Chen & H.M. Li EM620 (KUN), Chongqing, China, OR878464*, OR887615*, OR887625*; ***Stachysyingzuijieensis*** L. Wu & Y.P. Chen, L. Wu et al. YZJ0654 (CSFI), Hunnan, China, OR878471*, OR887622*, OR887613*; ***Stenogynebifida*** Hillebr., KF529652, KF235876, AF308221; ***Suzukialuchuensis*** Kudô, KF529653, MK909678, MK909744; ***Suzukiashikikunensis*** Kudô, KF529655, KF235889, KF235782; ***Thuspeinantabrahuica*** (Boiss.) Briq., KF529656, MK909679, MK909745; ***Thuspeinantapersica*** (Boiss.) Briq., KF529657, MK909681, MK909746.
